# Experimental and Theoretical Study of O-Substituent Effect on the Fluorescence of 8-Hydroxyquinoline

**DOI:** 10.3390/ijms16023804

**Published:** 2015-02-10

**Authors:** Mohie E. M. Zayed, Reda M. El-Shishtawy, Shaaban A. Elroby, Abdullah Y. Obaid, Zahra M. Al-amshany

**Affiliations:** 1 Chemistry Department, Faculty of Science, King Abdulaziz University, Jeddah B.O.208203, Saudi Arabia; E-Mails: mohiem@yahoo.com (M.E.M.Z.); skamel@kau.edu.sa (S.A.E.); aobaid@kau.edu.sa (A.Y.O.); zalamshany@hotmail.com (Z.M.A.); 2 Dyeing, Printing and Textile Auxiliaries Department, Textile Research Division, National Research Center, Dokki, Cairo 12622, Egypt; 3 Chemistry Department, Faculty of Science, Beni Suef University, Beni Suef 6251, Egypt

**Keywords:** ether and ester derivatives, 8-hydroxyquinoline (8-HQ), UV-visible and fluorescence spectra, TD-DFT calculations, electronic absorption, natural transition orbital (NTO)

## Abstract

The synthesis and characterization of different ether and ester derivatives of 8-hydroxyquinoline have been made. UV-visible and fluorescence spectra of these compounds have revealed spectral dependence on both solvent and O-substituent. The fluorescence intensity of ether derivatives revealed higher intensity for 8-octyloxyquinoline compared with 8-methoxyquinoline, whereas those of ester derivatives had less fluorescence than 8-hydroxyquinoline. Theoretical calculations based on Time-dependent density functional theory (TD-DFT) were carried out for the quinolin-8-yl benzoate(8-OateQ) compound to understand the effect of O-substituent on the electronic absorption of 8-hydroxyquinaline (8-HQ). The calculations revealed comparable results with those obtained from the experimental data. Optimized geometrical structure was calculated with DFT at B3LYP/6-311++G** level of theory. The results indicated that 8-OateQ is not a coplanar structure. The absorption spectra of the compound were computed in gas-phase and solvent using B3LYP and CAM-B3LYP methods with 6-311++G ** basis set. The agreement between calculated and experimental wavelengths was very good at CAM-B3LYP/6-311++G** level of theory.

## 1. Introduction

8-Hydroxyquinoline (8-HQ) and its derivatives, have a wide variety of important applications. For instance, they can be used as potential HIV-1 integrate inhibitors [1,2,3,4], and have antineoplastic [5,6] and herbicidal activity [6,7,8,9,10]. They are also used as preservatives in cosmetics and tobacco, chemical intermediates in dye synthesis as well as chromophoric and metallochromic indicators [11,12,13]. Compound 8-HQ has received continuous attention as a platform for the construction of a number of selective and efficient ionophores [14]. The most interesting feature of 8-HQ is its very sensitive fluorescence in aqueous or organic solutions but the fluorescence enhancement occurrs upon cation binding and many metal chelates of 8-HQ exhibit intense fluorescence [15,16,17,18,19]. Recent studies indicated that the on–off fluorescence of 8-HQ upon its interaction with metal ions has been attributed to a proton transfer mechanism in which 8-HQ is weakly or nonfluorescent (off) due to an excited-state proton transfer (ESPT); upon binding with the metal ion, fluorescence appears (on), due to the inhibition of the ESPT process [15,20]. Thus, it is anticipated that the replacement of hydrogen atom with different groups may lead to a better fluorescence compared with 8-HQ owing to the absence of the ESPT process.

On the other hand, interest in the calculation of electronic structures in excited states has been motivated by the increasing application of fluorescent molecules in a variety of research areas, including chemical biology, analytical chemistry, medicinal chemistry and molecular biology [21,22]. In recent years, calculations of electronic structures in the excited states have been a focus of interest because of the development of computations based on the time-dependent density functional theory (TDDFT) [23,24,25,26,27].

As a part of our work to achieve a priori prediction of excited states, we here compare the absorption energy calculated by the TDDFT approach. In addition, the solvent effect on the electronic absorption spectra is a useful tool to identify the electronic transitions of the molecules. This would help in studying the chemical properties of the excited states and to distinguish between the different electronic transitions. We will use the Continuum Polarizable model (PCM) [28,29].

Therefore, computational chemistry is thus necessary to gain insight into the molecular structure, although according to our best knowledge no evidence of similar study for the O-substituent effect on the fluorescence of 8-HQ has been reported in the chemical literature to date. In this work, interest resides in correlating the theoretically predicted electronic parameters with the accurate experimental results so as to provide possible explanations for the experimentally observed fluorescence-dependent O-substitution.

## 2. Results and Discussion

### 2.1. Synthesis

**Scheme 1:**
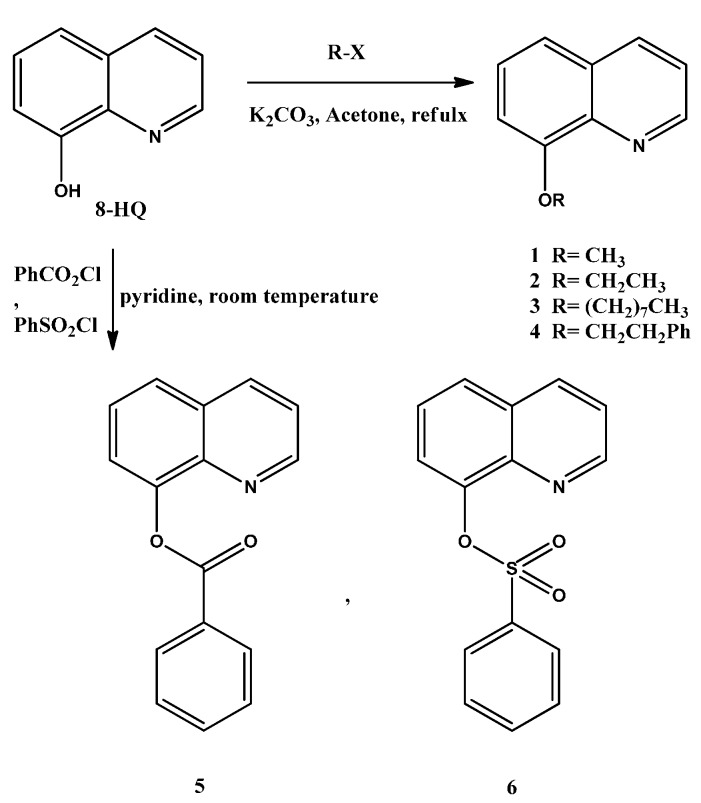
Synthesis of ether and ester derivatives of 8-HQ.

The 8-Alkoxyquinolines (**1**–**4**) and ester derivatives (**5**–**6**) were obtained by O-alkylation and O-acylationor O-sulfonylation in basic medium as shown in Scheme 1. The structure of these compounds was confirmed by ^1^H and ^13^C NMR, mass specrtometry and FTIR.

### 2.2. UV-Visible Spectra

Table 1 shows the solvent effect on the absorption and fluorescence properties of 8-HQ and its derivatives. Absorption and fluorescence spectra were recorded in MeOH, CH_3_CN and CHCl_3_. It is generally believed that the absorption spectra of 8-HQ is mainly attributed to its most stable form in which intramolecular hydrogen bonding is exist between N and H atoms [30,31].

**Table 1:** Solvent effects on the absorption and fluorescence properties of 8-HQ and its derivatives.

Compound	MeOH, K_T_(30) = 55.4		CH_3_CN, K_T_(30) = 45.6			CHCl_3_, K_T_(30) = 37.7	
	λ_max_, nm A	λ_max_, nm F	λ_max_, nm A	Molar Absorbivity, M^−1^·cm^−1^	λ_max_, nm F	λ_max_, nm A	λ_max_, nm F
8-HQ	315	-	316	10,200	400	311	-
8-MeQ	305	407	305	10,700	388	300	390
8-EtQ	306	405	302	13,900	392	307	392
8-PhetQ	312	406	304	31,700	387	305	387
8-OctQ	306	405	296	17,000	392	307	392
8-OateQ	283	-	276	22,400	385	279	393
8-SulfonateQ	274	359	273	19,200	392	275	397

K_T_(30) is the Dimroth solvent polarity index [32].

This intramolecular hydrogen bonding is sensitive to the solvent polarity and is stabilized in polar solvents. Compared with those obtained in MeOH and CH_3_CN, the absorption spectra of 8-HQ in CHCl_3_ is slightly blue shifted as CHCl_3_ is less polar than both MeOH and CH_3_CN. On the other hand and irrespective to the solvent type, small and a large blue shifts are observed when going from 8-HQ to its ether and ester derivatives, respectively. This blue shift is mainly attributed to the absence of intramolecular hydrogen bonding and to the respective structural difference as well.

### 2.3. Fluorescence Spectra

**Figure 1:**
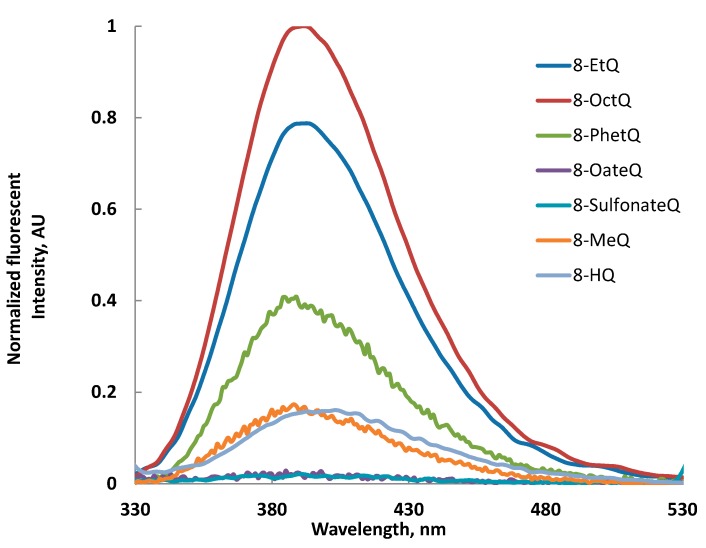
Normalized fluorescent intensity of different derivatives of 8-HQ in acetonitrile solution (1 × 10^−5^ M).

It has been reported that the weak fluorescence of 8-HQ in many solvents has been attributed to the excited state proton transfer (ESPT), therefore ether derivatives of 8-HQ would reveal higher fluorescence compared with 8-HQ. A red shift in the fluorescence of ether derivatives is observed in MeOH with compared values obtained in CH_3_CN and CHCl_3_. Also, the data shown in Table 1 reveals a negative solvatochromism, *i.e.*, blue shift on going from less polar solvent to higher one, for 8-SulfonateQ ester and almost no fluorescence for 8-OateQ ester. Figure 1 shows comparative normalized fluorescence intensity for 8-HQ and its ether and ester derivatives; the fluorescence intensity of 8-OctQ > 8-EtQ > 8-PhetQ > 8-MeQ ≥ 8-HQ was clearly observed.

**Figure 2:**
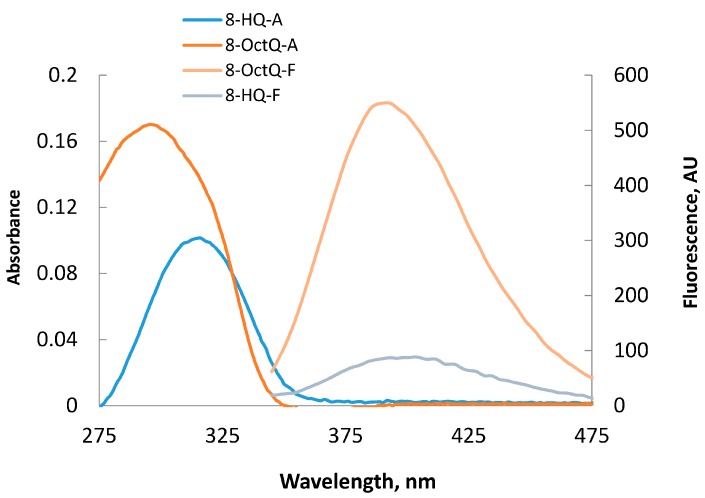
Absorption and fluorescence spectra of 8-HQ and 8-OctQ in acetonitrile solution (1 × 10^−5^ M).

This result reveals that the longer the alkyl chain, the higher the intensity of the fluorescence. Figure 2 shows a comparative fluorescence and UV-visible spectra of 8-OctQ and 8-HQ. The blue shift of the absorption and the florescence of 8-OctQ compared with 8-HQ is comparable with a large difference in the fluorescence intensity of 8-OctQ.

### 2.4. Molecular Orbital Calculations

**Figure 3:**
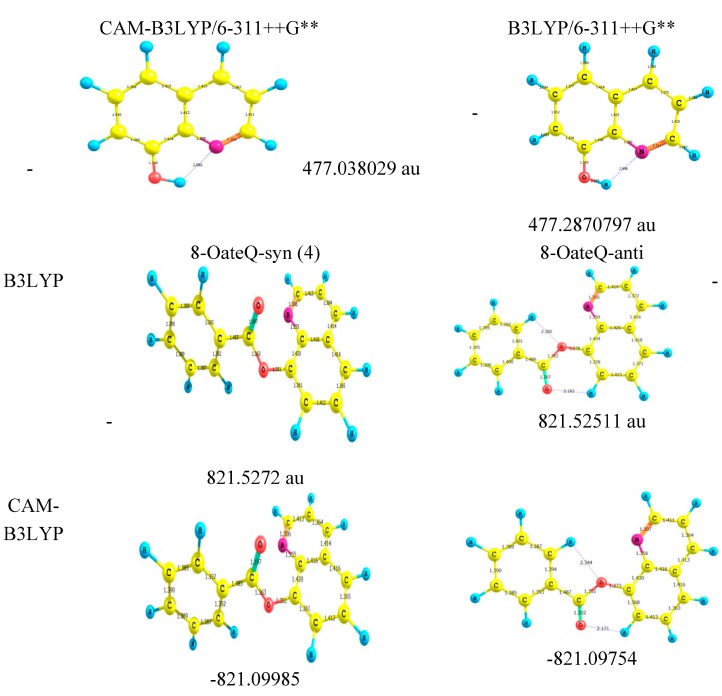
Relative energy and bond lengths for the 8HQ, 8-OateQ-syn and 8-OateQ-anti using B3LYP and CAM-B3LYP at 6-311++G** basis set.

A standard B3LYP hybrid functional and CAM-B3LYP were consistently employed for both geometry optimizations and TD-DFT calculations. The geometrical parameters (the bond lengths) and total energies for different conformers of 8-HQ and 8-OateQ using two different methods are summarized in Figure 3. The bond lengths in the two studied molecules are not different, and appreciably shorter than the standard single C–C bond length by c.a. 0.11 Å, which indicates moderate electronic resonance between the two moieties. Also, Figure 3 indicates clearly that 8-OateQ has two conformers. These two structures correspond to the syn and anti-conformers resulting from the rotation of the benzyl group around the ester single bond. The syn-conformer seems to be more stable by 1.311 kcal/mol to avoid the repulsion between lone pairs of oxygen and nitrogen atoms. This result is consistent with the experimental analysis.

Table 2, Table 3, and Table 4 show the evolution of λ_max_, molecular orbital contributions and oscillator strength of the studied molecules using TD-CAM-B3LYP and TD-B3LYP functionals in gas phase for 8-HQ and 8-OateQ. Details of TD-DFT calculations for 8-HQ have been reported in our previous work [15]. Theoretical calculations of 8-OateQ-syn spectra show two main band systems which are weak compared to 8-OateQ-anti. The second and third peaks centered at 268.28 nm (*f* = 0.008) and 266.68 nm (*f* = 0.026) with moderate to weak intensity, and a much stronger transition centered at 271.11 nm (*f* = 0.113). Moreover, the hypsochromic shift resulting from syn to anti isomerization is only very slightly underestimated about 10 nm, as shown in Table 3 and Table 4. On other hand, TD-CAM-B3LYP shows the stronger transition centered at 281.1 nm with large oscillator strength 0.226 for 8-OateQ-anti. These results are in a good agreement with the experimental data that indicates that the 8-OateQ excites in syn form with weak absorption and emission.

**Table 2:** TD-DFT spectral data of electronic transitions for 8HQ using CAM-B3LYP and B3LYP functionals at 6-311++G** basis set.

CAM-B3LYP/6-311++G**			
λ_max_	*f*	MO Contribution	MO Coefficient
302.49	0.06	38–39	0.70
-	-	37–40	0.11
275.58	0.002	38–40	0.53
248.83	0.003	36–39	0.69
B3LYP/6-311++G**			
339.88	0.037	38–39	0.69
288.49	0.004	38–40	0.55
265.57	0.002	36–39	0.7

**Table 3:** TD-DFT spectral data of electronic transitions for 8-OateQ-syn using CAM-B3LYP and B3LYP functionals at 6-311++G** basis set.

CAM-B3LYP/6-311++G**			
λ_max_	*f*	MO Contribution	MO Coefficient
271.11	0.113	65–66	0.63
-	-	61–66	0.19
268.28	0.153	61–66	0.50
-	-	64–66	0.36
-	-	65–68	0.29
266.68	0.003	65–66	0.41
B3LYP/6-311++G**			
292.04	0.084	65–66	0.64
-	-	61–66	0.2
-	-	62–66	0.14
288.4	0.14	61–66	0.57
-	-	62–66	0.37
-	-	64–66	0.17
277.02	0.002	65–67	0.69
-	-	60–67	0.11

**Table 4:** TD-DFT spectral data of electronic transitions for 8-OateQ-anti using CAM-B3LYP and B3LYP functionals at 6-311++G** basis set.

CAM-B3LYP/6-311++G**			
λ_max_	*f*	MO Contribution	MO Coefficient
281.21	0.225	65–66	0.66
-	-	64–68	0.13
268.94	0.01	64–66	0.47
268.80	0.002	61–66	060
B3LYP/6-311++G**			
307.96	0.0221	65–66	0.7
290.96	0.002	62–66	0.66
283.17	0.052	65–67	0.7

**Figure 4:**
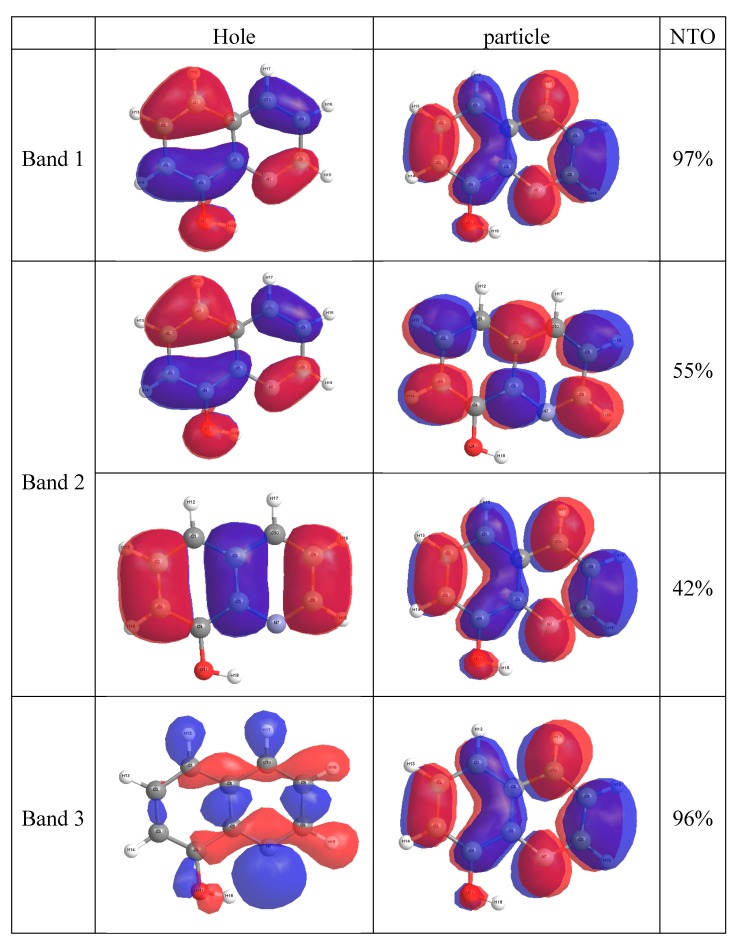
Natural Transition Orbital (NTO) for 8-HQ compound at CAM-B3LYP/6-311++G** level of theory.

**Figure 5:**
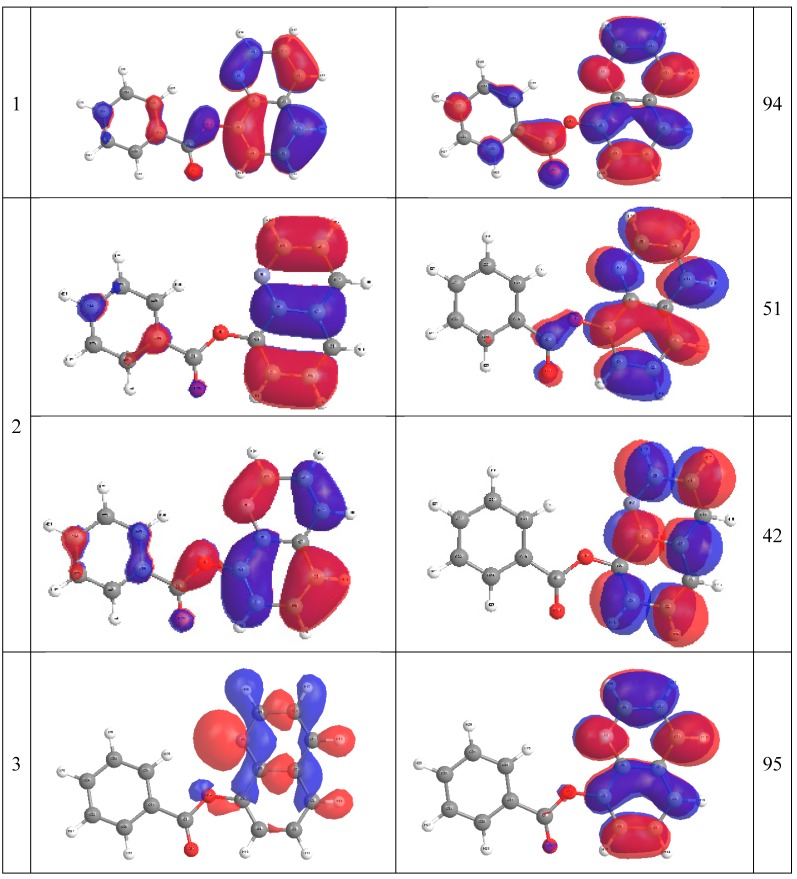
Natural Transition Orbital (NTO) for 8-OateQ-anti compound at CAM-B3LYP/6-311++G** level of theory.

**Figure 6:**
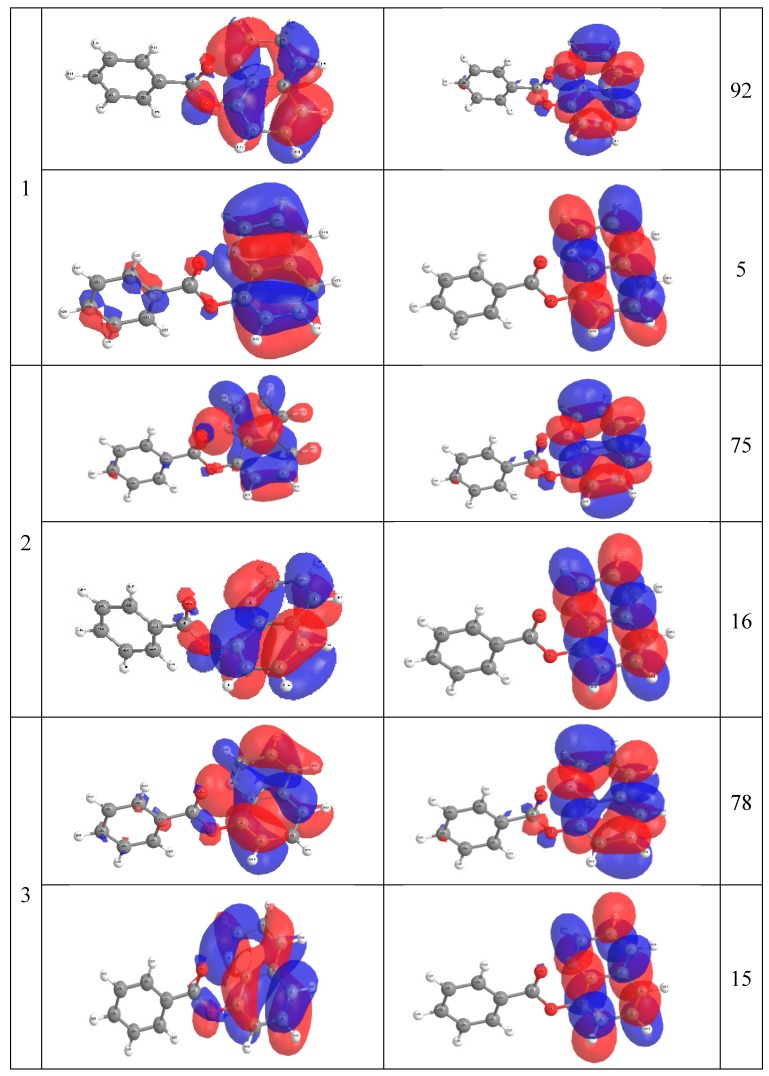
Natural transition orbital for 8-OateQ-syn compound at CAM-B3LYP/6-311++G** level of theory.

The comparison between the experimentally observed and theoretically computed spectra of 8-OateQ-in different solvents is summarized in Table 5. It should be noted that CAM-B3LYP reproduced the experimental spectra compared to B3LYP method for this system where the calculated λ_max_ using CAM-B3LYP is very close to experimental values. For solvated 8-OateQ, there is a nice agreement between experimental and theoretical λ_max_ for both anti and syn forms, especially in acetonitrile and chloroform; the differences do not exceed 6.0 nm. In further detail concerning the electronic structure, we characterized the low-lying three singlet excited states within the current TD-CAM-B3LYP scheme as in Figure 4, Figure 5 and Figure 6. Figure 4, Figure 5 and Figure 6 show the main orbitals that contributed in the vertical electronic transitions in the studied molecules, which were calculated using TD-CAM-B3LYP/6-311++G** level of theory. In case of the parent compound (8-HQ), the first and second peaks originate from the π–π* intramolecular Charge transfer (CT) transitions from phenol moiety to pyridine ring. The first intense peak, dominantly observed with HOMO–LUMO excitation, has a large *f* of 0.06. As can be seen in Figure 3 and Figure 4, the HOMO and LUMO of anti and syn are very similar. In the case of 8-OateQ-anti, all transition originates from HOMO (mainly distributed on the Q moiety) to LUMO with a large *f* of 0.226. The results indicated that the benzoate group does not have any contribution on the absorption. The theroretical calculations are in good agreement with the experimental data as shown in Table 5.

**Table 5:** Comparison between theoretical CAM-B3LYP/6-311++G** and experimental wavelength for the studied species in different solvents.

8-HQ		
Methanol	Acetonitrile	Chloroform
296.78	296.84	299.31
273.47	273.47	274.17
246.66	246.67	247.39
**Experimental**		
315	316	311
**8-OateQ-syn**		
**Methanol**	**Acetonitrile**	**Chloroform**
270.96	271.03	271.83
267.11	267.13	267.55
259.75	259.73	261.79
**Experimental**		
314	276	279
**8-OateQ-anti**		
**Methanol**	**Acetonitrile**	**Chloroform**
282.03	282.12	283.31
268.83	268.39	268.78
262.87	262.86	264.43
**Experimental**		
314	276	279

## 3. Experimental Section

### 3.1. General

All solvents and reagents were of the highest purity available, purchased from Sigma–Aldrich Company (Seelze, Germany) and used as received. ^1^H and ^13^C NMR spectra were recorded in CDCl_3_ solutions on a Bruker Avance 600 MHz spectrometer. Infrared spectra were performed on a PerkinElmer spectrum 100 FTIR spectrometer. Mass spectra were measured on a GCMS-QP1000 EX spectrometer at 70 eV. UV-visible absorption spectra were recorded with a Jasco V560 spectrophotometer (Jasco international Co., Ltd., Tokyo, Japan). Fluorescence spectra were conducted on a Perkin-Elmer LS-55 Luminescence Spectrometer and uncorrected. Melting points were determined in open capillary tubes in a Stuart Scientific melting point apparatus SMP3 and are uncorrected.

### 3.2. Synthesis

#### 3.2.1. Etherification, General Procedure

8-Hydroxyquinaline (1.46 g, 10 mmol), alkyl or aryl halide (15 mmol) and potassium carbonate (2.76 g, 20 mmol) were added to acetone (50 mL). The mixture was stirred at reflux for 8 h. The mixture was then filtered off and the residue was washed three times with acetone. After removal of the solvent, the residue was purified by column chromatography using ethylacetae/petroleum ether (2:8) as the eluent to afford the corresponding ether product in good yield.

##### 8-Methoxyquinoline (1)

Oil.^1^H NMR (CDCl_3_, 600 MHz, ppm): δ 4.14 (s, 3H, OCH_3_), 7.16 (d, 1H, *J* = 7.8 Hz, CH-Ar), 7.47 (d, 1H, *J* = 7.8 Hz, CH-Ar), 7.56–7.60 (m, 2H, 2CH-Ar), 8.33 (dd, 1H, *J* = 8.1 Hz, 1.2 Hz, CH-Ar), 9.07 (dd, 1H, *J* = 4.5 Hz, 1.2 Hz, CH-Ar). ^13^C NMR (CDCl_3_, 150 MHz, ppm): δ 56.21, 108.82, 119.41, 121.70, 127.82, 129.45, 138.51, 147.81, 154.10. IR (cm^−1^): 3060, 2936, 2838, 1616, 1597, 1571, 1500, 1473, 1377, 1316, 1261, 1194, 1109, 1076. Calc. for C_15_H_11_NO_3_S: 159.19 [M]^+^; Found: 159.1 [M]^+^.

##### 8-Ethoxyquinoline (2)

Oil.^1^H NMR (CDCl_3_, 600 MHz, ppm): δ 1.64 (t, 3H, *J* = 13.8 Hz, CH_3_), 4.34 (q, 2H, *J* = 13.8 Hz, OCH_2_), 7.07 (d, 1H, *J* = 7.8 Hz, CH-Ar), 7.39 (d, 1H, *J* = 8.4 Hz, CH-Ar), 7.44–7.49 (m, 2H, 2CH-Ar), 8.17 (d, 1H, *J* = 8.4 Hz, CH-Ar), 8.99 (dd, 1H, *J* = 4.2 Hz, 1.2 Hz, CH-Ar). ^13^C NMR (CDCl_3_, 150 MHz, ppm): δ 14.66, 64.58, 109.17, 119.24, 121.53, 127.42, 129. 51, 137.61, 138.40, 148.32, 153.8. IR (cm^−1^): 3040, 2980, 2925, 1614, 1597, 1570, 1501, 1468, 1377, 1316, 1257, 1102. MS (*m*/*z*): Calc. for C_11_H_11_NO: 173.22 [M]^+^; Found: 173.1 [M]^+^.

##### 8-(Octyloxy)quinoline (3)

Oil.^1^H NMR (CDCl_3_, 600 MHz, ppm): δ 88 (t, 3H, *J* = 7.2 Hz, CH_3_), 1.24–1.42 (m, 10H, CH_2_), 1.53 (quin, 2H, *J* = 7.2 Hz, CH_2_), 2.04 (quin, 2H, *J* = 7.2 Hz, CH_2_), 4.43(t, 2H, *J* = 7.2 Hz, OCH_2_), 7.07 (d, 1H, *J* = 7.2 Hz, CH-Ar), 7.39 (dd, 1H, *J* = 7.8 Hz, 1.2 Hz, CH-Ar), 7.43–7.49 (m, 2H, 2CH-Ar), 8.17 (dd, 1H, *J* = 7.8 Hz, 1.2 Hz, CH-Ar), 9.98 (dd, 1H, *J* = 4.2 Hz, 1.8 Hz, CH-Ar). ^13^C NMR (CDCl_3_, 150 MHz, ppm): δ 14.13, 22.68, 26.07, 28.93, 29.26, 29.42, 31.85, 69.27, 109.21, 119.25, 121.55, 127.32, 129.57, 137.32, 138.84, 148.54, 154.21. IR (cm^−1^): 3038, 2924, 2855, 1615, 1597, 1570, 1500, 1465, 1376, 1317, 1261, 1105. MS (*m*/*z*): Calc. for C_17_H_23_NO: 257.38 [M]^+^; Found: 257.1 [M]^+^.

##### 8-Phenethoxyquinoline (4)

Oil.^1^H NMR (CDCl_3_, 600MHz, ppm): δ 3.37 (t, 2H, *J* = 7.8 Hz, CH_2_), 4.43 (t, 2H, *J* = 7.8 Hz, OCH_2_), 7.24–7.44 (m, 4H, 8CH-Ar), 8.12 (dd, 1H, *J* = 8.4 Hz, 1.8 Hz, CH-Ar), 8.96 (dd, 1H, *J* = 4.2 Hz, 1.8 Hz, CH-Ar). ^13^C NMR (CDCl_3_, 150 MHz, ppm): δ 35.63, 69.75, 108.75, 119.7, 121.6, 126.62, 126.65, 128.61, 129.12, 129.52, 135.92, 137.77, 140.34, 149.37, 154.51. MS (*m*/*z*): Calc. for C_17_H_15_NO: 249.32 [M]^+^; Found: 249.1 [M]^+^.

#### 3.2.2. Esterification, General Procedure

8-Hydroxyquinaline (1.46 g, 10 mmol), benzoly chloride or benzene sulfonyl chloride (10 mmol) and pyridine (0.79 g, 10 mmol) were added to acetone (50 mL). The mixture was stirred at room temperature overnight. The mixture was then filtered off and the residue was washed three times with acetone. After removal of the solvent, the residue was recrystallised from ethanol to afford the corresponding ester product in good yield.

##### Quinolin-8-yl benzoate (5)

Solid, m.p = 112–114 °C. ^1^H NMR (CDCl_3_, 600 MHz, ppm): δ 7.43 (dd, 1H, *J* = 8.4 Hz, 4.2 Hz, CH-Ar), 7.53–7.6 (m, 4H, 4CH-Ar), 7.66 (t, 1H, *J* = 7.2 Hz, CH-Ar), 7.77 (dd, 1H, *J* = 7.5 Hz, 1.8 Hz, CH-Ar), 8.21 (dd, 1H, *J* = 8.4 Hz, 1.2 Hz, CH-Ar), 8.36 (m, 2H, 2CH-Ar), 8.90 (dd, 1H, *J* = 4.2 Hz, 1.8 Hz, CH-Ar). ^13^C NMR (CDCl_3_, 150 MHz, ppm): δ 121.63, 121.74, 126.02, 126.25, 128.58, 129.50, 129.6, 130.58, 133.58, 135.99, 141.43, 147.74, 150.64, 165.51. IR (cm^−1^): 3062, 1731, 1596, 1582, 1500, 1471, 1450, 1388, 1316, 1267, 1255, 1230, 1164, 1096. MS (*m*/*z*): Calc. for C_16_H_11_NO_2_: 249.27 [M]^+^; Found: 249.1 [M]^+^.

##### Quinolin-8-yl benzenesulfonate (6)

Solid, m.p = 104–106 °C.^1^H NMR (CDCl_3_, 600 MHz, ppm): δ 7.38 (dd, 1H, *J* = 8.4 Hz, 4.2 Hz, CH-Ar), 7.46 (t, 2H, *J* = 7.8 Hz, 2CH-Ar), 7.51 (t, 1H, *J* = 7.8 Hz, CH-Ar), 7.59 (t, 1H, *J* = 7.8 Hz, CH-Ar), 7.62 (d, 1H, *J* = 7.8 Hz, CH-Ar), 7.75 (d, 1H, *J* = 8.4 Hz, CH-Ar), 7.99 (d, 1H, *J* = 7.8Hz, CH-Ar), 8.12 (d, 1H, *J* = 8.4Hz, CH-Ar), 8.78 (d, 1H, *J* = 4.2 Hz, CH-Ar). ^13^C NMR (CDCl_3_, 150 MHz, ppm): δ 121.86, 122.67, 126.03, 127.07, 128.77, 128.80, 129.61, 133.92, 135.71, 136.14, 141.52, 145.47, 150.74. IR (cm^−1^): 3050, 1593, 1494, 1466, 1449, 1370, 1312, 1230, 1185, 1158, 1070. Calc. for C_15_H_11_NO_3_S: 285.32 [M]^+^; Found: 285.1 [M]^+^.

### 3.3. Computational Methods

B3LYP/6-311++G** [30,31,33,34] level of theory was used to calculate the electronic properties of the title molecules in this work. All calculations were performed using Gaussian 09W program package [31]. The equilibrium geometry corresponding to the true minimum on the potential energy surface (PES) has been obtained by frequency calculation. TD-DFT was used to determine the excitation energies asking for the lowest three singlet-singlet excitation energies on the B3LYP-optimized geometry. In the TD-DFT calculations, B3LYP and CAM-B3LYP were employed to validate the functionals. In TD-DFT calculations, solvent effects of ethanol, acetonitrile and methanol were included using the Polarizable Continuum Model (PCM). The default Gaussian09 PCM implementation (non-equilibrium formulation) is suitably designed to predict UV/Vis spectrum described within the vertical transition scheme, where solvent polarization responds to the change of electronic distribution of the excited state molecules with the molecular orientation fixed during electronic transition.

## 4. Conclusions

In this paper, different ether and ester derivatives of 8-hydroxyquinoline have been synthesized. Compared with ester derivatives, ether derivatives of these compounds revealed higher fluorescence intensity and the highest intensity value was obtained using 8-octyloxyquinoline. UV-visible absorption maxima of the 8-hydroxyquinoline and its derivatives were examined experimentally as well as computationally with combinations of 6-311++G** basis sets and B3LYP and CAM-B3LYP methods in the gas phase and solvent. Theoretical calculations using CAM-B3LYP/6-311++G** level of theory provide a good description of positions of the one band maximum in the observed spectrum. The geometries of a series of 8-HQ and 8-OateQ are reproduced using two different DFT methods. The calculated energy data at these computational levels show that the non-planar structure of 8-OateQ is more stable than the planar one.
